# Detecting trends and shocks in terrorist activities

**DOI:** 10.1371/journal.pone.0291514

**Published:** 2023-09-15

**Authors:** Rafael Prieto-Curiel, Olivier Walther, Ewan Davies

**Affiliations:** 1 Complexity Science Hub, Vienna, Austria; 2 Department of Geography, University of Florida, Gainesville, Florida, United States of America; 3 Radcliffe Observatory Quarter, Mathematical Institute, University of Oxford, Oxford, United Kingdom; National Institute of Genomic Medicine: Instituto Nacional de Medicina Genomica, MEXICO

## Abstract

Although there are some techniques for dealing with sparse and concentrated discrete data, standard time-series analyses appear ill-suited to understanding the temporal patterns of terrorist attacks due to the sparsity of the events. This article addresses these issues by proposing a novel technique for analysing low-frequency temporal events, such as terrorism, based on their cumulative curve and corresponding gradients. Using an iterative algorithm based on a piecewise linear function, our technique detects trends and shocks observed in the events associated with terrorist groups that would not necessarily be visible using other methods. The analysis leverages disaggregated data on political violence from the Armed Conflict Location & Event Data Project (ACLED) to analyse the intensity of the two most violent terrorist organisations in Africa: Boko Haram (including its splinter group, the Islamic State West Africa Province), and Al-Shabaab. Our method detects moments when terrorist groups change their capabilities to conduct daily attacks and, by taking into account the directionality of attacks, highlights major changes in the government’s strategies. Results suggest that security policies have largely failed to reduce both groups’ forces and restore stability.

## Introduction

The temporal distribution of attacks committed by terrorist organisations varies enormously, depending on internal and external factors. Changes in leadership and strategy, military interventions or even domestic politics can all increase or decrease the *intensity* or the daily number of attacks attributed to a group. However, the fluctuations of terrorist events complicate the analysis of a group and make it difficult for analysts to detect if the rate is increasing or decreasing. More severe events intuitively require more planning, so observing periods with low activity of a terrorist group might not indicate that its power has been reduced [1]. Further, it has been observed that the severity of terrorist events, as well as the time between consecutive events, has certain regularities, often described by a power-law distribution [2, 3], a property that was also observed for war casualties [4], and other social events [5]. Terrorist events tend to be irregularly distributed during the year, with periods of relative calm followed by massive waves of attacks [6], making security policies particularly challenging to implement in the long run. For this reason, detecting whether the changes observed in the intensity of terrorism violence represent a progressive increase or are caused by internal and external shocks remains a crucial and long-lasting issue. In other terms, are terrorist organisations continuously increasing the frequency of their attacks, or are they adopting a more opportunistic strategy, striking when the moment is most favourable?

Although there are some techniques for dealing with sparse and concentrated discrete data, standard time-series analyses appear ill-suited to understanding the temporal patterns of terrorist attacks due to the sparsity of the events. For example, data collected by the Armed Conflict Location & Event Data project (ACLED) [7] shows that Jihadists part of the Boko Haram insurgency in northern Nigeria were active 272 days of the year and involved in 613 events in 2018, killing nearly 3,000 individuals. If we let *X*_*i*_ be the number of Boko Haram events in day *i* in 2018, most values of *X*_*i*_ are either zero or one. There is no significant trend in that series, meaning we cannot recognise if the group is less active by the end of the year. There are no weekly cycles, indicating that the group is not statistically more active on Wednesdays or Tuesdays, for example. Also, the correlation between *X*_*i*_ and *X*_*i*+1_ is not statistically significant, meaning that a day with high activity is not necessarily followed by a day with high (or low) activity. Many time-series techniques do not provide significant results concerning the count of events.

Altering the time units with smaller or larger periods is hardly a solution. On the one hand, refining daily intervals into shorter periods would make the time series even more sparse and, therefore, more challenging to obtain relevant information or trends. Unlike financial time series, where it is possible to get specific values for different moments or interpolate them, terrorism events can hardly be observed in short intervals. The data, after all, is a discrete count of events. On the other hand, aggregating observations into weekly or monthly events could show structural changes and cycles, but with the cost of reducing 365 data points into only 52 weeks or 12 months. Further, events grouped in arbitrarily large periods are often not comparable: the number of events per month ignores that January has 10% more days than February or that one month might have five full weekends and another month only four, which could be relevant if some group was less active during weekends.

Another challenge is the directionality of an event. There are two types of events when a terrorist group is involved: the attacks conducted by the terrorist group and the response by government forces, victims and society. Although events committed against a group are not terrorist events, they are crucial to understanding how they react to their circumstances. For example, roughly 20% of the events in which Boko Haram was involved in 2019 were coordinated by military forces against the group, causing nearly 30% of the casualties related to Boko Haram in that year. Frequently, studies on terrorism focus only on the violent events committed by the group without considering how the state forces and society will respond. However, with that procedure, it is impossible to detect if a strategy prevents more violence or if a terrorist event was triggered as retaliation against some counter-terrorism attack. On the contrary, considering all events in which a terrorist group is involved without distinguishing directionality is equally problematic. In that case, it is impossible to determine whether an increase in activity results from a group becoming more violent or because security forces increasingly target this group.

We propose a novel technique for analysing low-frequency temporal events, such as terrorism and its lethality. The method is based on the cumulative curve and its corresponding gradients. We estimate the daily intensity of events by modelling the cumulative curve of events. Our approach assumes that events are a point process resulting from a background distribution with a time-varying rate. Observing the cumulative distribution of events allows us to analyse events, independently of how rare, sparse and concentrated they are, and to identify major events and shocks that correspond to either internal changes in the structure of a terrorist organisation or external shocks, such as counter-terrorism initiatives. We primarily apply our technique to the Jihadist group known as Boko Haram and its splinter group, the Islamic State West Africa Province (ISWAP), which has killed an average of ten people daily since the late 2000s and contributed to displacing 2.4 million people in West Africa [8]. We then classify events as being committed by or against Boko Haram and measure the daily rate and shocks of both time series, detecting significant events that could have triggered them. Using an open-source dataset that monitors political violence’s evolution since 1997, we generalise our findings to another Jihadist organisation, Al-Shabaab, which has conducted thousands of deadly attacks in the Horn of Africa for over 15 years.

## Theoretical background

The causes that explain why people turn to terrorism defy broad generalisations [9]. Grievances, religious faith, or the desire for change combine in different ways and to varying degrees in each conflict rather than operate as universally deterministic causes [10]. Even more importantly, some factors routinely used to describe the process of radicalisation have very little explanatory power: most people do not turn to terrorism because they are poor, uneducated, or brainwashed, for example [11, 12].

Numerous theories have been developed to capture this complexity, focusing on strategic, psychological, or organisational factors [13]. The concepts developed within each theory often reflect disciplinary choices, with the strategic model built on rational choice theory being the dominant model in political science [14], and the model built on personality and mental issues more predominant in psychology [15]. In network science and related disciplines, the organisational structure of terrorist groups and their relationship with the broader society remain the main focus of analysis [16]. Unlike other approaches that focus on social inequalities, poverty, education, or natural resources to explain terrorism, this meso-level approach is particularly well adapted to examine how the social structure of terrorist groups can help understand their political emergence and spatial diffusion, possibly across ethnic groups and countries [17]. The organisational approach argues that joining a terrorist organisation is a decision to give individual and collective meaning. In West Africa, for example, some women have joined Boko Haram because the Nigerian military had killed their husband or child, but also because they see joining the jihadist group as the only way to emancipate themselves from traditional laws [18].

In the last two decades, quantitative studies have made considerable progress in visualising and modelling this meso-level. Today, agent-based models help detect patterns that emerge when hundreds or thousands of events are observed [19, 20]. Advances in network analysis have also contributed to mapping the social structure of terrorist organisations [21–26]. Novel techniques have been developed to help detect future attacks [27], including the use of mobility patterns [28] and early warnings systems [29]. These techniques complement geo-visualising tools that can track terrorist organisations and help identify regularities in their spatial patterns [30, 31].

### Time series

Recent advances have also been made in understanding the temporal dimension of terrorism and the corresponding trends of terrorist attacks [32], a crucial dimension that complements the analysis of terrorist social and spatial patterns [33]. Detecting trends and cycles in terrorism can help identify shifts caused by anti-terrorist operations and internal struggles between factions [34, 35]. Such detection can be done by looking at the correlation between different types of attacks [36] or time intervals between consecutive events [37]. Time series analysis can be used in terrorism analysis [38, 39] to build auto-regressive models [40], observe self-exciting processes [6, 41–43], make predictions [44], and measure some of the impacts of terrorist attacks [45, 46]. Based on terrorism time series, it has been observed that the time between consecutive events is heavy-tailed [1, 47], meaning that significant periods of inactivity are frequently observed.

Time series often display an abrupt variation due to a transition between states, known as change points. Detecting change points is a powerful technique applied in terrorism studies [48, 49]. In general, detecting change points is based on dividing a series into distinct homogeneous segments, observing the probability distributions before and after some point, and identifying it as a “shock” if the two distributions are significantly different [50, 51]. However, although time series analysis is a powerful tool in terrorism studies, certain aspects of terrorism data considerably differ from the most common time series applications. Financial time series analysis, for instance, is based on a metric *μ* at a specific time *t*, say *μ*_*t*_, such as the value of a stock or the exchange rate between two currencies. It is possible to model the data based on some smoothing technique, such as a moving average, splines, or a kernel density estimate [52, 53], and analyse the seasonality, long-term trends, and other temporal variations [54] such as weekly fluctuations [52]. Kalman filters and correlations with other series can also be calculated to understand the data structure and its noise or predict future values [55]. In time series, where *μ*_*t*_ indicates the value of the series at that specific moment, it is possible to differentiate it or to refine the time partition by considering not only the daily exchange rate but its hourly values or even smaller time intervals.

However, in the case of terrorism, there are some challenges to applying time series techniques. The most relevant challenge is that terrorism time series corresponds to counts of events that are relatively rare, sparse and concentrated, meaning that observations have to be grouped into long intervals (such as weeks or even months) to give insightful information. Besides that, precisely identifying all the events is often impossible to obtain. For large terrorist organisations, some events are missing, whereas others might wrongly be associated with the group. Periods with low activity can indicate that a terrorist group is losing its capabilities or, instead, planning for more severe attacks [1]. Finally, events in which terrorist groups are involved have two other critical elements. One is its severity, and the other one is its directionality. In terms of severity, some events could be relatively minor (if it has no casualties, no injured people and no infrastructure is damaged), but some could be highly severe. In terms of directionality, some events are committed by a terrorist group, but the group suffers from other events, losing members and resources. Thus, an increase in the number of events between state forces and a terrorist organisation can either be interpreted as a sign that the government is progressively dismantling its opponents or, on the contrary, losing ground when faced with a powerful non-state actor.

Despite these challenges, some progress has been made to incorporate temporal data-driven models and detect shocks and pulses in similar time series. In the case of terrorism, a shifting intensity has been used to model a self-exciting terrorist activity [6], detect the factors which alter terrorist groups’ failure [56–58], or analyse the duration of peacetime periods between consecutive attacks [59], based on the premise that the daily intensity should observe shifts after major and significant changes in the group or its environment.

## Methods

Our study focuses mainly on the Boko Haram insurgency active in the Lake Chad region, divided between Nigeria, Niger, Cameroon and Chad in West Africa. Boko Haram and ISWAP are currently among the deadliest armed organisations in Sub-Saharan Africa, with more than 40,000 casualties recorded since the organisation became violent in 2009 [60]. Military offensives against Boko Haram and ISWAP have claimed the lives of 26,000 soldiers and insurgents in the transnational region in the last decade [8].

Our study leverages data from the Armed Conflict Location & Event Data project (ACLED) [7]. It monitors political violence worldwide, mainly from local and regional media, reports from NGOs and social media accounts. ACLED is the most comprehensive, up-to-date, and spatially detailed conflict database. Its event-based data include the location, type, number of fatalities, and actors involved in violent events. From 2000 to 2020, ACLED reported 689,625 events, resulting in more than one million deaths. In line with previous studies on the geography of armed groups in Africa [8, 28], our paper focuses on three types of violent events in which Boko Haram is mainly represented: battles (violent interactions between armed groups, including government forces); violence against civilians; and remote violence (including grenades, airstrikes, missiles and other remote systems).

All events in which one of the factions of Boko Haram was involved as an *actor* or *associated actor* were selected from the ACLED dataset, including the faction commonly known as Boko Haram or Jama’at Alhul Sunnah Lidda’wati wal Jihad, led by Abubakar Shekau, and its splinter group ISWAP led by Abu Mus’ab Al-Barnawi (both leaders were killed in 2021). The data comprises 5,048 events with more than 42,000 fatalities, with an average of 1.28 events daily. We classify the events being committed *by* or *against* Boko Haram and/or ISWAP by looking at the type of event and its text description. We analyse the daily intensity separately. From 1 January 2010 to 20 March 2020, there was a varying rate of events and casualties of Boko Haram and ISWAP (Table 1). In 2015, Boko Haram insurgents were involved in nearly 32 daily casualties, and each event caused an average of 18 casualties. By 2019 the groups were involved in 29% more events than in 2015, but each event was 80% less lethal. In so many years, it is clear that Boko Haram insurgents have changed their strategy and updated their objectives and methods. However, exactly when and why is unclear by simply looking at the aggregated yearly data.

**Table 1:** Daily number of Boko Haram and ISWAP events (*e*), their casualties (*c*) and the ratio of casualties per event (*c*/*e*) between 1 January 2010 and 20 March 2020.

year	2010	2011	2012	2013	2014	2015	2016	2017	2018	2019	2020
*e*	0.09	0.34	1.10	0.82	1.53	1.77	1.50	1.86	1.68	2.28	3.67
*c*	0.21	1.73	5.27	8.25	26.54	31.88	9.90	10.39	8.16	8.36	10.13
*c*/*e*	2.20	5.14	4.79	10.10	17.39	18.02	6.59	5.58	4.86	3.66	2.76

To generalise our findings to other regions of the African continent, we also apply our method to the Al-Shabaab terrorist organisation, principally active in Somalia and Kenya. We identify Al-Shabaab events following the same strategy, giving us 13,295 events with nearly 33,000 casualties between 2006 and 20 March 2020 (Table 2).

**Table 2:** Daily number of Al-Shabaab events (*e*), their casualties (*c*) and the ratio of casualties per event (*c*/*e*) between 1 January 2010 and 20 March 2020.

year	2010	2011	2012	2013	2014	2015	2016	2017	2018	2019	2020
*e*	1.42	1.70	2.87	3.28	3.99	3.37	3.64	5.07	4.58	3.90	4.06
*c*	5.48	3.50	6.74	5.47	9.31	8.81	12.12	12.87	11.09	7.79	5.49
*c*/*e*	3.85	2.06	2.35	1.67	2.33	2.61	3.33	2.54	2.42	1.99	1.35

### Abrupt changes and a stepwise intensity of events

The date, number of casualties, and type of event are considered for each event. The data consists of an ordered sequence of a finite number of *n* events, *e*_1_, *e*_2_, …, *e*_*n*_, which correspond to the time at which each event occurs. From the *n* events, we construct the series *y*_*k*_ as the cumulative number of events which have occurred before time *τ*_*k*_ as

$$\begin{array}{c} y_{k}=\sum_{j=1}^{n}\;H\left(\tau_{k}-e_{j}\right), \end{array}$$ (1)

where the function *H*(*x*) is the Heaviside step function, where *H*(*x*) = 1 if *x* ≥ 0 and zero otherwise. The series *y*_1_, *y*_2_, …, *y*_*m*_ consists of *m* observations corresponding to the cumulative number of events on *m* days. The objective is to approximate the series *y*_*k*_ by a piecewise linear function and analyse the linear pieces and their gradients and shifts.

Consider the expected cumulative curve of events up to a specific time, assuming some background rate of events. Formally, let *C*(*t*) be the expected cumulative number of events *C* of a specific type, from time *t*_0_ = 0 until time *t* > 0 in days. We model the daily number of events as an inhomogeneous point process with a time-varying intensity function λ(*t*). That is, we assume that the number of events on the day *t* is a point process with a time-varying rate λ(*t*). The underlying Poisson distribution of the point process is frequently used to model discrete counts of events, such as incorrect criminal verdict [61], casualties in interstate wars [62], armed conflicts [63], gang-related violence [64], or the impact of incarceration [65].

Our approach assumes that events are a point process resulting from a background distribution with a time-varying rate. We assume that the underlying distribution of the point process is Poisson, meaning that the number of events for the day *t*_*j*_ follows a distribution *Po*(λ(*t*_*j*_)). The expected number of events on a day *t*_*j*_ is given by λ(*t*_*j*_), meaning that the number can be directly interpreted for the distribution. Further, the distribution can be considered for more extended periods. For example, for a set of days, say [*t*_*j*_, *t*_*j*+1_, …, *t*_*j*+*k*_] the expected number of events is given by $\sum_{u=0}^{k}\lambda \left(t_{j+u}\right)$, so it provides an easy approximation for the expected number of events for longer intervals. A point process with a time-varying rate as the background intensity function is frequently used to model crime counts [66, 67] since it ignores small fluctuations but focuses on the daily rate λ(*t*). The function λ(*t*) ≥ 0 is known as the “intensity” of the events, and it is interpreted as the expected number of daily events in which a terrorist organisation takes part after the small fluctuations are removed. Thus, it permits us to observe the general evolution of violence instead of daily oscillations, which are often misleading.

Here, we are interested in abrupt changes in the intensity function λ(*t*). The intensity λ(*t*) is modelled as a stepwise function with multiple change points [68], so we assume that it is constant for some a set of days and then shifts from λ_1_ to λ_2_ at a certain break, and then shifts to λ_3_ and so on. Each λ_*i*_ corresponds to the daily rate, and the breaks correspond to events which alter the organisation’s capacity. The rate λ(*t*) is a step function. Let 0 = *t*_1_ ≤ *t* < *t*_2_ be the first interval, such that we can express λ(*t*) = λ_1_ ≥ 0. For *t*_2_ ≤ *t* < *t*_3_, we express λ(*t*) = λ_2_ ≥ 0 and so on for each interval. The expected number of cumulative events *C*(*t*) is obtained as

$$\begin{array}{c} C\left(t\right)=\int_{0}^{t}\;\lambda \left(s\right)\;ds. \end{array}$$ (2)

That function can be expressed as a continuous piecewise linear non-decreasing function, for *t* ≥ 0 as

$$\begin{array}{c} C\left(t\right)=\sum_{j=1}^{\kappa }\;\beta_{j}\left(t-t_{j}\right)H\left(t-t_{j}\right), \end{array}$$ (3)

where *κ* is the number of intervals, and where the coefficients are defined by λ_1_ = *β*_1_, λ_2_ = *β*_1_ + *β*_2_, and in general λ_*j*_ = *β*_1_ + *β*_2_ + ⋯ + *β*_*j*_.

It is possible to model the number of events on a single day using other discrete distributions, such as a Binomial, Negative Binomial or even a Multinomial distribution. However, they require fixing some parameters, such as the maximum number of events on a single day. The Poisson model depends on a single parameter that matches the expected number of events on that day, so its interpretation is straightforward. It is also possible to extend the Poisson distribution to model events that trigger the occurrence of further events. A self–exciting process is based on the idea that the rate of events λ(*t*) can be modelled depending on the events that have occurred up to time *t*. A self-exciting process is one type of extension to the Poisson model where the function λ(*t*) is assumed to jump after the occurrence of an event, with a subsequent cooling down process, capturing some retaliation. This model has been used to model burglaries, gang violence, and terrorist events [6, 27, 36, 64, 69, 70].

### Parameter estimation

For parameter estimation, the first step is to detect if a homogeneous -constant- rate is sufficient for explaining the observed data. During *m* days, there were *n* events, so the rate λ_0_ = *n*/*m* is the estimated daily rate of events with no jumps or breaks. Based on this rate, it is possible to simulate the number of events. For each day, a Poisson distribution is considered *S*_*i*_ *Po*(λ_0_) reflecting the possible number of events under a homogeneous rate. The simulated cumulative number of events *S*_1_(*t*) = ∑_*i*≤*t*_ *S*_*i*_ then is computed. In our case, the random sampling process is repeated a sufficiently large number of times, fixed at 100 repetitions. An arbitrarily large number, such as 100, is frequently used for simulating a point process to produce possible extreme values and intervals. A similar type of test, also using 100 repetitions, is frequently used to detect if the distribution of a spatial point process corresponds to one with a homogeneous rate [71]. With this process, we obtain the simulated number of events *S*_1_(*t*), *S*_2_(*t*), …, *S*_100_(*t*). For day *t*, we have 100 observations reflecting the possible number of events that could be observed under a homogeneous rate. We drop the top and bottom two observations to remove possible simulation outliers. We compute the lower bound, defined as *S*_*l*_(*t*) = min_*i*_ *S*_*i*_(*t*) and the upper bound, defined as *S*_*u*_(*t*) = max_*i*_ *S*_*i*_(*t*). These two simulated observations capture departures expected in the cumulative number of events assuming the homogeneous rate λ_0_. We then compare the simulated bounds [*S*_*u*_(*t*), *S*_*l*_(*t*)] against the observed one *C*(*t*). If *C*(*t*) is outside the [*S*_*u*_(*t*), *S*_*l*_(*t*)] for some days, then we reject a homogeneous rate.

Having rejected a homogeneous rate, the procedure then is to estimate those varying rates. We follow an iterative process to estimate the daily rate of events with an unknown number of breaks. We start with *κ* = 1 breaking point and estimate the rate of events assuming that the data has one point in the first observation (so the first day of the data). Then we iteratively add one extra breaking point and estimate the daily rate of events with *κ* breaking points. For *κ* breaking points, we need to estimate their location (the day of the intensity shifts) and the rates between each shift. The expected number of cumulative events *C*(*t*) depends on the location of such breakpoints 0 = *t*_1_, *t*_2_, …, *t*_*κ*+1_ and the gradient parameters *β*_1_, *β*_2_, …, *β*_*κ*_. They are obtained by minimising the Sum of Squares Error (SSE) between the observed number of cumulative events *y*_*k*_ and the expected one *C*(*t*_*k*_) as

$$\begin{array}{c} SSE=\left(2\kappa +1\right)\sum_{k=1}^{m}{\left(y_{k}-C\left(t_{k}\right)\right)}^{2}, \end{array}$$ (4)

where 2*κ*+1 is the number of parameters estimated. The parameters *β*_*i*_ are derived as a piecewise linear regression, using a package for segmented relationships in regression models called *Segmented* [72, 73]. This method implements a form of gradient descent to minimise the *SSE* concerning the breaking points and the rates.

With *κ* = 1, the intensity of events is constant, and we get that λ(*t*) = *β*_1_ = *n*/*m*. This way, the expected number of events after *m* days is exactly *n*, so *C*(*t*) = *β*_1_*t*, for *t* ≥ 0, where *β*_1_ is the daily speed at which events are accumulated. The rate *β*_1_ is the expected number of events per day. With *κ* = 2 there is a breaking point in *t*_2_, such that *C*(*t*) = *β*_1_*t* + *β*_2_(*t* − *t*_2_)*H*(*t* − *t*_2_), which means that for values of *t* in the [0, *t*_1_) interval, the speed is *β*_1_ and for values of *t* ≥ *t*_1_, we get that *C*(*t*) = (*β*_1_ + *β*_2_)*t* − *β*_2_*t*_1_, so that the speed of events, after *t*_2_ is (*β*_1_ + *β*_2_). For more intervals, with *κ* > 2, the piecewise speed is given by $\sum_{j=1}^{s}\beta_{j}$ for *t* ∈ (*t*_*s*_, *t*_*s*+1_). Eq 3 gives a continuous non-decreasing function, where λ_*j*_ = *β*_1_ + *β*_2_ + ⋯ + *β*_*j*_, so that at all times, the speed of events stays non-negative.

The number of breaks on the series is unknown, and, as with many other data techniques, no rule applies to all cases [50]. In general, by adding more breaks, intervals between consecutive shocks get narrower and, eventually, too thin to be considered a trend. Thus, for the application of terrorism data, we assume that a shock and a change in the daily rate of events should be observed for roughly one month to be considered a shock. Thus, we stop the iterative procedure when any interval contains less than 30 days. It depends on the type of data considered and its length: 3,935 days of data are available for Boko Haram against 4,955 days for Al-Shabaab. Therefore, with our 30-day rule, we finish the procedure with 13 breaks for Boko Haram and 16 breaks for Al-Shabaab.

We combine the rates obtained for different breaking points into a single rate for each day by considering the mean and lower and upper bounds to obtain departures of the daily rate. Formally, for day *t*, the obtained rates for *s* breakpoints λ^(*s*)^(*t*), for *s* = 1, 2, 3, …, *κ*, are considered. We define the rate as $\lambda \left(t\right)=\bar{\lambda }\left(\tau_{k}\right)$, and upper and lower values give us the corresponding intervals. The daily rate λ(*t*) is the expected number of daily events in which a terrorist organisation participates.

The daily rate λ(*t*) combines piecewise linear functions. The values obtained for the daily rate λ(*k*) help us construct intervals for the number of observed events. For day *k*, we simulate a point process *s*_*k*_ for the number of events, and then, we consider the cumulative number of events $c_{k}=\sum_{j=1}^{k}s_{j}$. Following the same procedure a sufficiently large number of times (in our case, set to 100) and removing the simulated outliers, we obtain an interval for the expected number of cumulative events by day *k*. The principle is the same as the one applied to reject a homogeneous rate. Instead of a unique value of λ_0_, we use the varying λ(*t*). The obtained intervals capture the departures and variability given our estimated daily rate.

The λ(*t*) is a step function with up to *κ*(*κ* + 1)/2 breaks. However, some of those shocks might reveal almost negligible shifts in the daily rate, whilst some might show drastic changes in the before and after rates. Our method for detecting shocks from a series of events is based on the cumulative sum of the distribution and observing when the cumulative sum departures exceed a specified threshold [74]. We say that a *shock* happened at some time *τ* if |λ(*τ* + 7) − λ(*τ*)| > 1.2λ(*τ*), meaning that the rate varied by more than 20% in less than one week, and the events near those dates identify the shocks. Although our definition of a shock is based on the arbitrary 20% increase or decrease in the rate, it results in a manageable number of events to interpret. This method’s effectiveness depends on the data being considered, particularly the length of the period, the number of events, and their variability.

**Fig 1:**
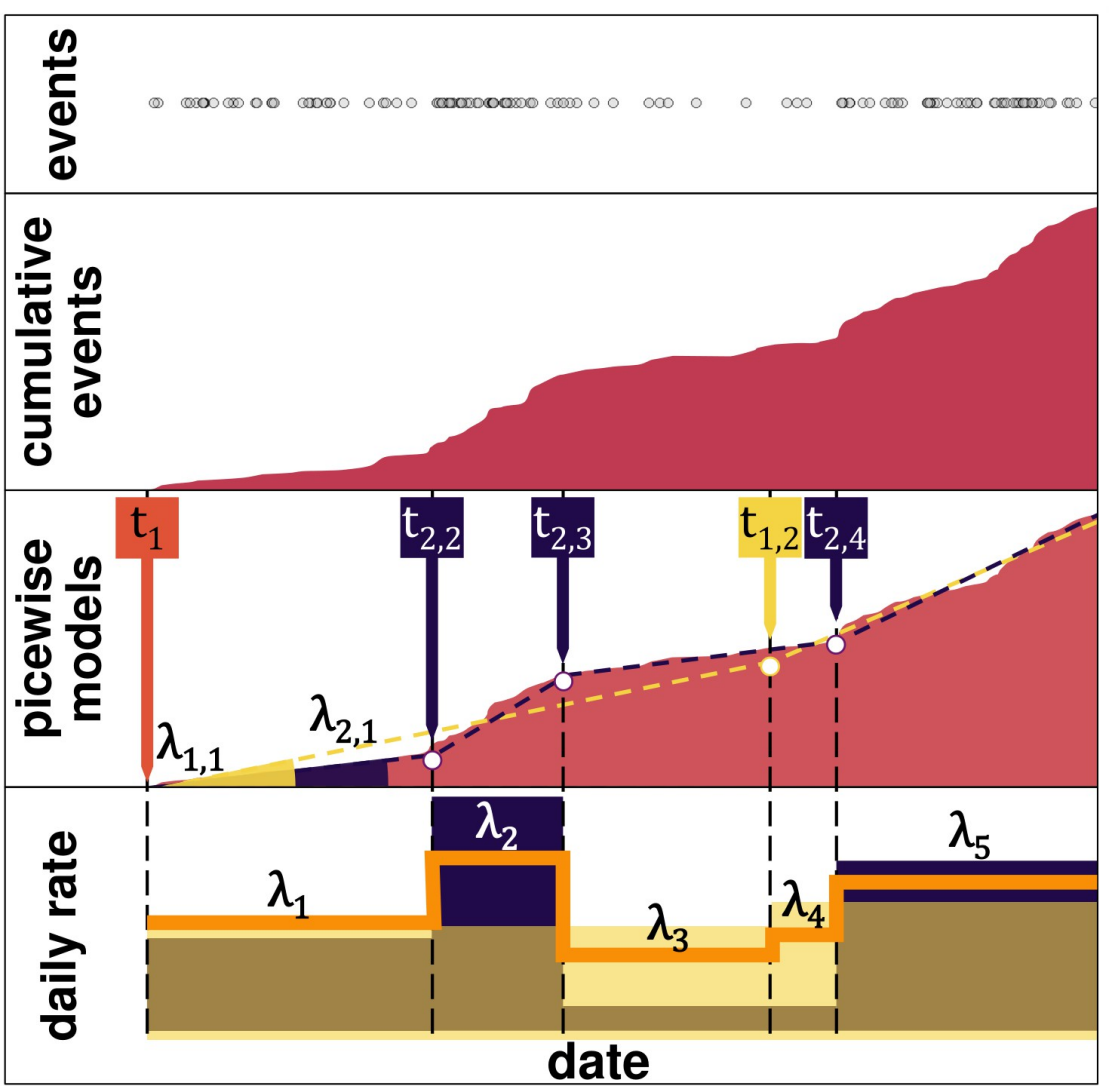
Model based on the cumulative number of events. The number of events in a certain time interval, plotted as marks in the top part, forms the cumulative number of events (red polygon in the middle part). The cumulative curve is estimated by a piecewise linear function with a specific number of breaks (the yellow and the purple polygons in the middle part, with one and three breaks as an example). Each section’s gradient is considered the organisation’s daily rate from the piecewise linear function, represented as the yellow and purple polygons in the bottom part of the figure. The corresponding rates are combined (below) into the average rate (orange line) for each day in the analysis.

In sum, we use the cumulative curve as a series of *n* observations, representing the total number of events before each day. First, we test whether a homogeneous rate is sufficient for explaining the fluctuation of events. If the homogeneous rate is rejected, a constant rate of events is insufficient to explain the data, so we estimate a time-varying rate. The cumulative curve is then approximated by a piecewise linear function, considering an increasing number of breaking points. The daily rates for a different number of breaking points are combined by computing the mean considering intervals. It is possible to simulate the number of events for each day from the daily rates. Then, shocks are obtained by detecting moments where the daily rate varies drastically over a short period (Fig 1).

The obtained daily rates inform a single day’s expected number of events. Rather than detecting a single day with many events, our method picks on substantial and sustained changes in the daily rate. Shocks are detected and analysed based on the political and social events that could have caused an increase or decrease in the rate of terrorist events around those dates. Events are filtered using the corresponding labels to analyse other aspects, such as the specialisation or the objectives. The cumulative curves and daily rates are constructed following the same procedure.

### From the observed data to the daily rate

**Fig 2:**
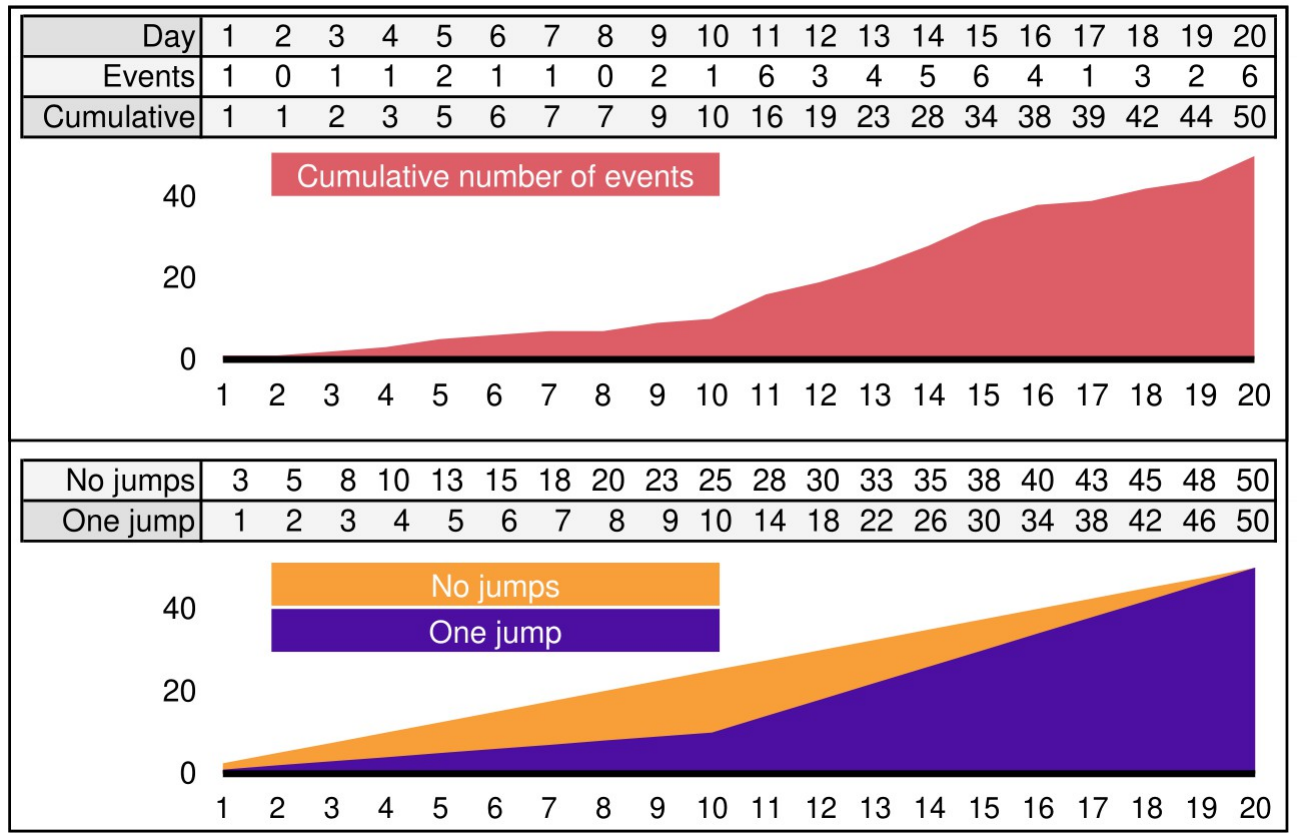
Simulated series obtained from a simulated point process. The number of events in the first ten days is sampled with a rate λ_1−10_ = 1, and then, from days 11 to 20, they are sampled with rate λ_11−20_ = 4. A shock between days 10 and 11 is observed in the cumulative polygon (top) and the estimated rates (bottom).

A simple example shows how to estimate rates from some data. Fig 2 has 20 days of simulated data. The first ten days were obtained from a point process with a rate λ_1_ = 1 and the next ten days with a rate λ_2_ = 4, so we expect 50 events. We observe fluctuations in the daily number of events, even with a fixed rate. For instance, there are zero or two events during the first ten days even if the rate is λ_1_ = 1. The cumulative number of events is then constructed. The change in the daily rate is quite noticeable. Before day ten, for every *k* days, we expect to accumulate *k* events, but after day ten, for every *j* days, we expect 4*j* events.

Assuming no rate jumps, the estimated intensity gives $\hat{\lambda }=5/2$. This estimation (orange polygon) does not resemble the observed data well (it misses the break in the middle). We add one break to the estimation (purple polygon) and obtain that the SSE drops from 1364 to 153. There seemingly is a third jump around day 16. Due to randomness, days 11 to 16 seem to have a higher intensity than the subsequent days. We obtain ${\hat{\lambda }}_{11-16}=4.6$ between days 11 and 16 and a rate of ${\hat{\lambda }}_{17-20}=3$ from days 17 and 20 with the value of the SSE going from 153 to 100. With four breaks, the SSE does not decrease further, so we stop with three breaks. Notice that the jump in day 16 is only due to randomness. In general, this is the case since the background rates are unknown. With an unknown number of jumps, we average the rate for each day by considering all values obtained for a different number of jumps. In the example, by taking the average with up to two jumps, we get an estimated rate of ${\hat{\lambda }}_{1-10}=1.5$ between days 1 and 10; an estimated rate of ${\hat{\lambda }}_{11-16}=3.7$ between days 11 and 16, and an estimated rate of ${\hat{\lambda }}_{17-20}=3.2$ for days 17 to 20. We report a shock on day ten only since the jump in day 16 alters the rate $\hat{\lambda }$ by less than 20%.

## Results

### Boko Haram events

**Fig 3:**
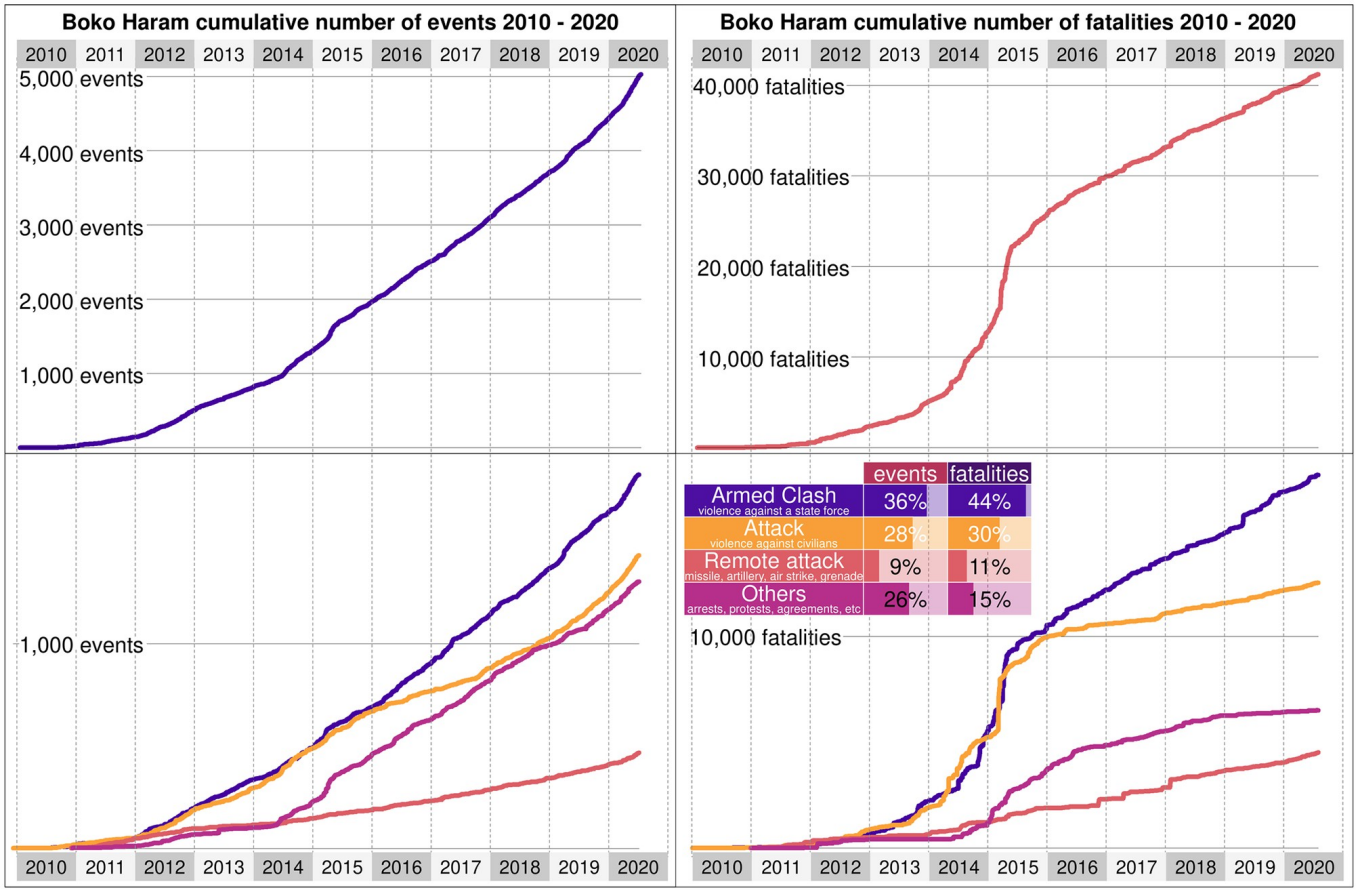
Boko Haram and ISWAP events. Left—Cumulative number of events (vertical axis) in which Boko Haram and ISWAP have participated since 2010 (horizontal axis), including both events by Boko Haram and ISWAP and against them. The top panel shows all events. The bottom panel shows events by type, including battles, violence against civilians, remote violence, and other events, such as protests or arrests. Right—Cumulative number of fatalities (vertical axis) in which Boko Haram and ISWAP have participated since 2010 (horizontal axis), including both events by Boko Haram and ISWAP and against them. The top panel shows all fatalities. The bottom panel shows events by type, including battles, violence against civilians, remote violence, and other events, such as protests or arrests.

First, we test a homogeneous rate. For Boko Haram, we observe that λ_0_ = 1.28, so if events followed a homogeneous rate, we would expect 1.28 each day. By March 24 2014, there were 950 events, while with a homogeneous rate, we would have expected between [*S*_*u*_(*t*), *S*_*l*_(*t*)] = [2112, 2355]. Therefore, we reject a homogeneous rate. We proceed with the stepwise model. The cumulative number of events at the top of Fig 3 suggests that Boko Haram and ISWAP experienced prolonged periods when they could produce roughly the same impact daily (straight lines), for example, from 2016 to 2018. The figure also suggests several breaking points during which the cumulative distribution of events is no longer linear, as in 2012 or 2020. One can assume that these breaks correspond to changes in leadership between competing factions or intense pressure from counter-insurgency forces that have forced Boko Haram and ISWAP to continuously adapt their military strategy and tactics. The bottom of the figure represents the same evolution by disaggregating violent events into three categories: battles, violence against civilians, and remote violence.

The data shows that until early 2015, the number of battles and attacks against civilians increased roughly at the same speed. This changed rapidly after the Nigerian military and the Multinational Joint Task Force (MNJTF) launched a series of counter-offensives that contributed to weakening Boko Haram and accelerated internal divisions within the organisation. In March 2015, Boko Haram pledged allegiance to the Islamic State during the worst possible moment for the organisation. Shortly afterwards, Boko Haram splits into two competing factions, one led by Abubakar Shekhau and the other by Abu Mus’ab Al-Barnawi, known today as ISWAP. These events led to a temporary decrease in the number of attacks against civilians and a growing number of armed clashes between government and insurgent forces, which exceeded the number of attacks against civilians until the end of 2017. The increasing gap between the two types of attacks during this period suggests that Boko Haram and ISWAP were increasingly fighting a coordinated military force. The number of events that led the government to regain territory, shown at the bottom of Fig 3, increased accordingly. Since 2018, attacks against civilians have resumed and expanded considerably.

**Fig 4:**
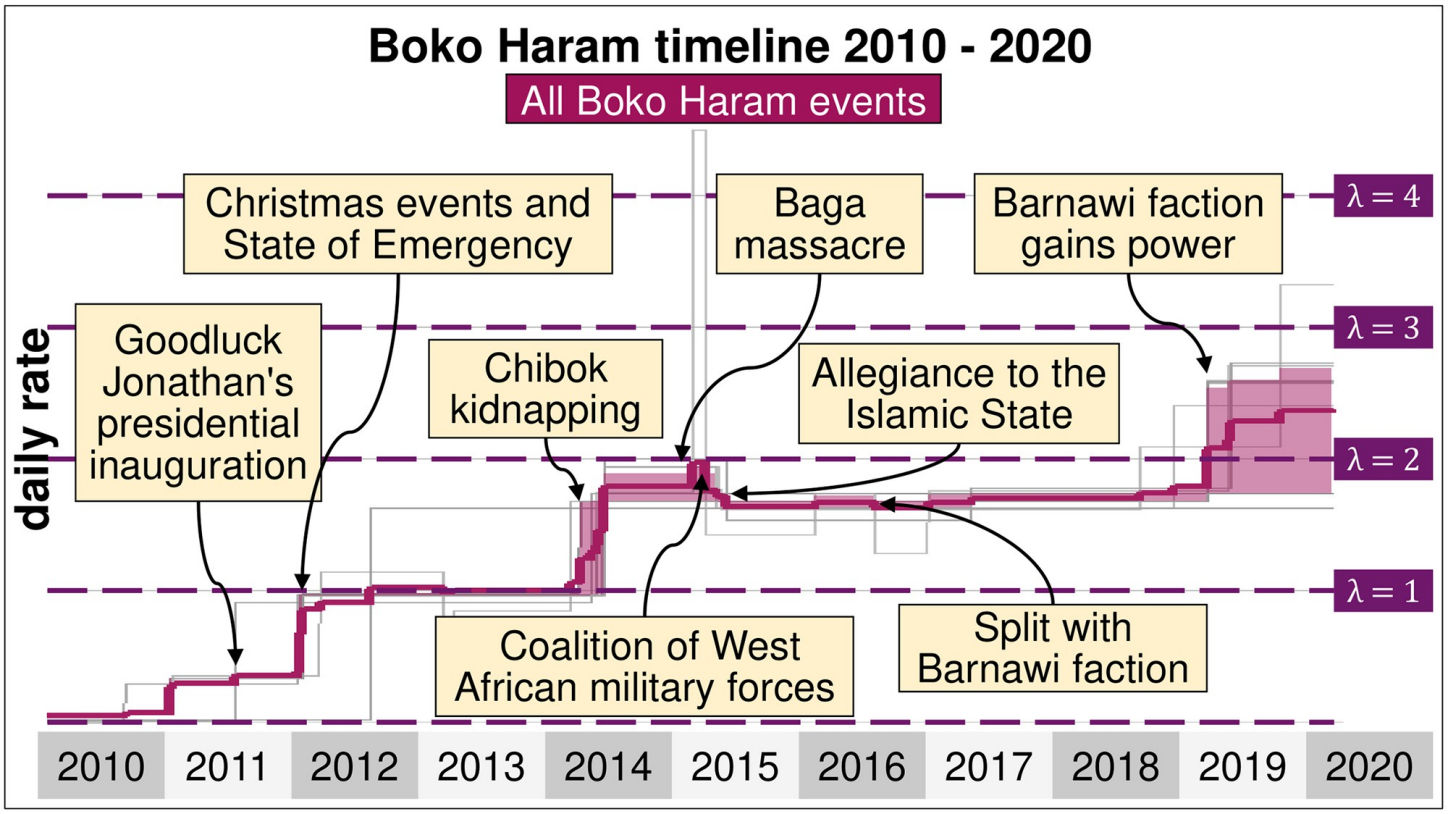
Estimated daily intensity of events in which Boko Haram and ISWAP take part between January 2010 and March 2020. A daily rate of λ = 1 means that the organisation is expected to commit one event each day, although there is a fluctuation around the number. It excludes protests, agreements, non-violent transfers of territory and other non-violent events.

A total of 12 breaking points can be observed in the Boko Haram time series for nearly 4,000 days of data, corresponding to 13 different segments from January 2010 to March 2020. Some of the breaking points correspond to rapid changes in event rates and can be classified as shocks (Fig 4). The first shock was observed in the first week of November 2010. Before, Boko Haram took part in roughly one event every 16 days, but it took part roughly in one event roughly every 3 to 4 days, becoming a pervasive problem in Nigeria. A few months later, a second shock was observed in April 2011, a few days after Goodluck Jonathan won the presidential election. The daily rate of events went from roughly one every three days (so λ = 1/3) to one every day (so λ = 1). Boko Haram started a bombing campaign that targeted the Nigerian police and United Nations in the federal capital of Abuja before launching a series of deadly attacks against Christian churches. In December 2011, President Jonathan declared a state of emergency in the northern part of the country and shut land borders with Cameroon, Chad and Niger to prevent militants’ movement to neighbouring countries. These measures were followed by a period of relative calm, with some shocks recorded in the daily rate of events between 2012 and 2013, such as the April 2013 Baga massacre, whose responsibility is disputed.

The next major shock occurred in early 2014 when the daily rate of events nearly doubled. This period corresponds to a surge in Boko Haram activities, particularly against civilians, who were more regularly killed or kidnapped by the insurgents [75] and by increasing clashes between Boko Haram, government forces and allied militias, such as the Civilian Joint Task Force (CJTF). The Nigerian government launched a series of military operations against Boko Haram, without much success, due to the inadequate training, corruption and lack of equipment of the Nigerian army.

In 2015, Boko Haram reached its most significant territorial expansion and committed numerous massacres in Borno State that culminated with the killing of up to 2000 civilians in the city of Baga and the destruction of the headquarters of the MNJTF in January. The Nigerian military responded to the degradation of the security situation with a large-scale offensive launched under the umbrella of the MNJTF. Forces from Chad, Cameroon, and Niger supported the Nigerian military in a series of coordinated attacks that inflicted heavy losses on Boko Haram and forced the organisation to relocate to remote areas such as the Mandara mountains of Cameroon or the islands of Lake Chad. The last major shock occurred in 2019 when the insurgents regained power in the countryside, from which the military retreated.

### Directionality of events

Thus far, our analysis has considered all violent events in which Boko Haram and ISWAP had been involved, either as perpetrators or as targets of attacks. However, an overall increase in the number of attacks associated with one organisation can either mean that it is becoming more dangerous or that the government is increasingly targeting it. For this reason, distinguishing between perpetrators and targets of a terrorist incident is crucial to understanding long-term shocks and trends. In what follows, we analyse the notes associated with each event to determine the *directionality* of each attack recorded since 2010. For each event, we determine if it was committed *by* Boko Haram, ISWAP and their adversaries or *against* them. For example, if ACLED mentioned that *Boko Haram members attack civilians on the island of Boram*, we considered that Boko Haram was responsible for the attack. If, on the contrary, ACLED mentioned that *Military forces launched a raid on a Boko Haram hideout in Bulabulim*, we assumed that the event was committed against Boko Haram. Civilians were always considered victims of violent events.

The results show that Boko Haram and ISWAP were responsible for 69.5% of the 5,048 events observed in the region. Military, police and state forces were accountable for 24.6% of the events. Other actors, including vigilantes, initiated 1.1% of the events (Table 3). In some cases, we were unable to determine the directionality of attacks. This is the case for several battles between military forces and Boko Haram. For example, if ACLED mentioned that *Boko Haram and military forces clashed in Amchide*, both actors can be seen as perpetrators or victims of the event. These incidents, representing only 6.7% of the events, were classified in a separate category.

**Table 3:** Frequency of events committed by each group.

Actor	% of events
Boko Haram and ISWAP	69.5%
Military Forces of Nigeria	16.7%
MNJTF	2.2%
Other military forces	3.2%
Police forces	0.7%
Other actors and vigilantes	1.1%
Battles without clear perpetrators	6.7%
Total	100%

**Fig 5:**
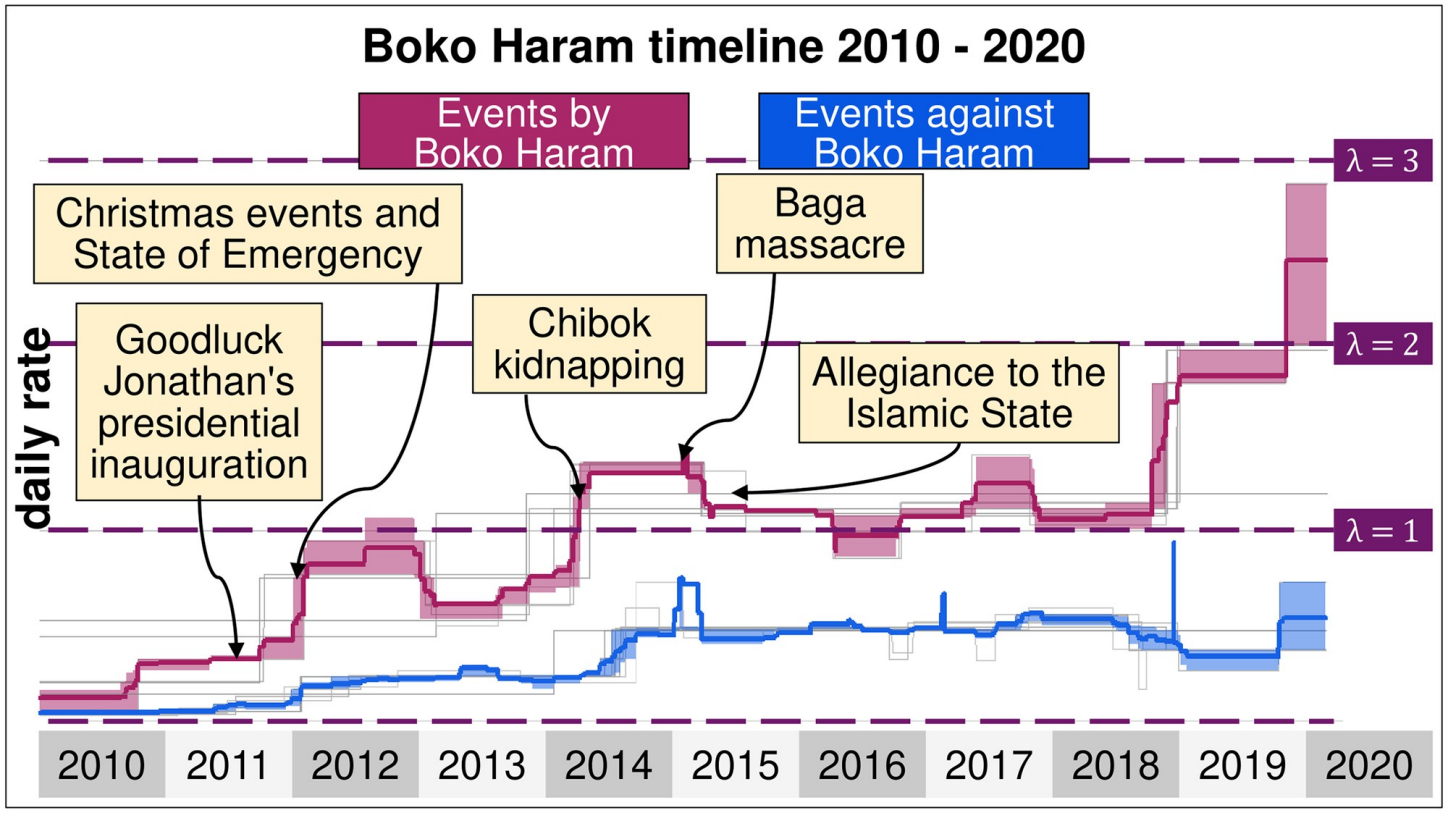
Rate of events by and against Boko Haram. Daily rate in which Boko Haram and ISWAP commit an event (in red) and daily rate of events against Boko Haram and ISWAP (in blue) between 2010 and 2020.

The directionality of attacks allows us to understand better the evolution of the daily rate of attacks of Boko Haram and ISWAP (Fig 5). Generally speaking, while Boko Haram and ISWAP experience significant fluctuations in the number of events committed against their common enemies, government forces retaliate at a considerably lower rate. The daily rate of events committed by Boko Haram and ISWAP was nearly 0 in 2010 and increased rapidly over the decade, reaching one daily event in 2018 (λ_2018_ = 1). Since 2018, the daily rate of attacks by Boko Haram and ISWAP has more than doubled, while the rate of events against the two Jihadist organisations has remained stable (λ = 0.5). These findings suggest that Boko Haram and its splinter group are primarily responsible for intensifying the northern conflict and that government forces are mainly unable to cope with the steady increase in attacks.

**Fig 6:**
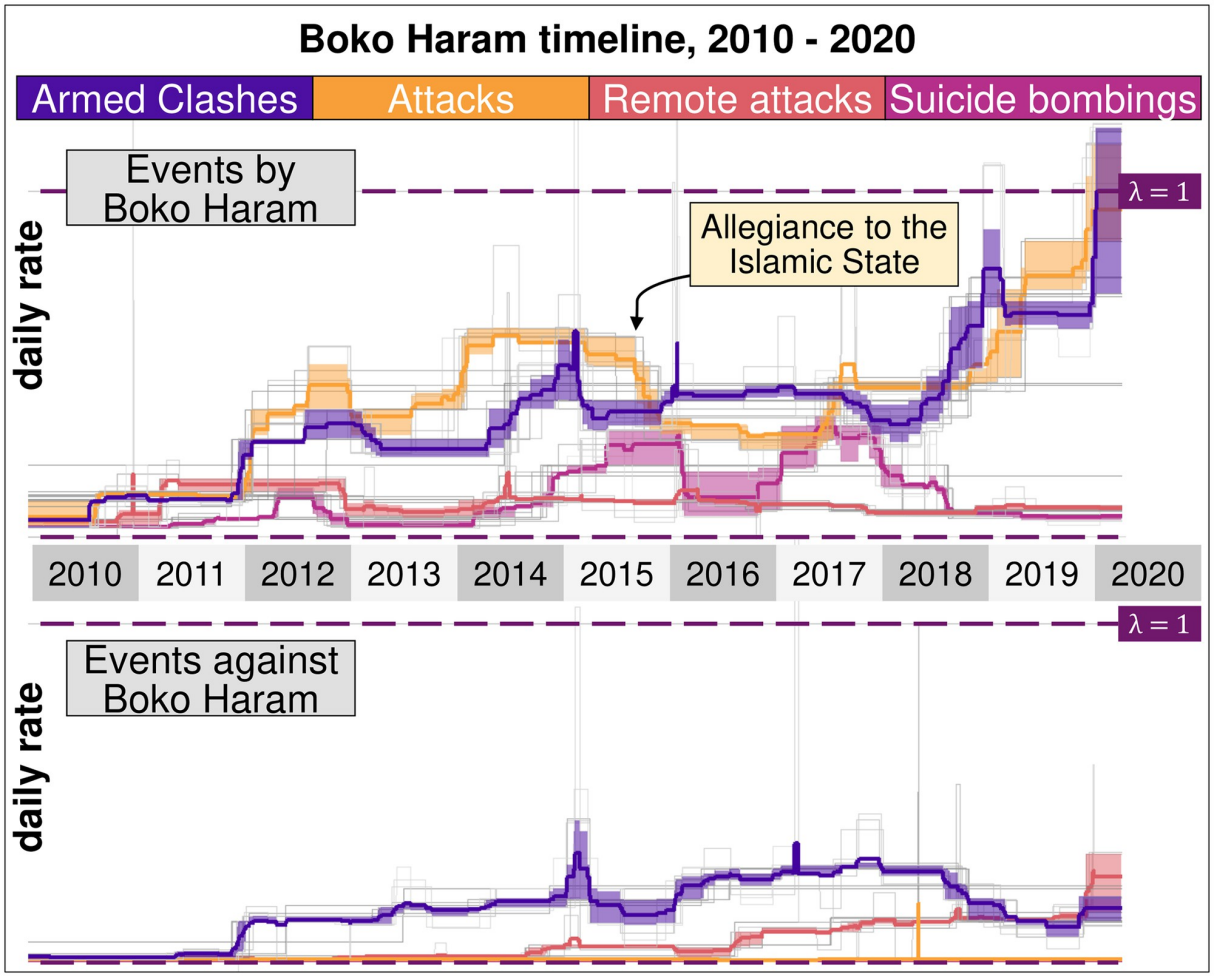
Rate of different types of events by Boko Haram. Daily rates in which Boko Haram takes part in different types of events, including attacks against civilians, against a state force, remote attacks and suicide bombings, between 2010 and 2020. The top panel is the events committed by Boko Haram and ISWAP, and the bottom panel is the events against the terrorist organisation. Suicide bombings are codified as Explosions and Remote Violence by ACLED.

The cumulative curve of events (Fig 5) highlights some crucial breakpoints in the speed of accumulation of events, particularly when it comes to attacks against civilians, as during the Christmas Events (2012), the Chibok kidnapping (2014), or the Baga massacre (2015). These key events become even more visible when the data is disaggregated by event types (Fig 6). Both figures suggest that Boko Haram and ISWAP have targeted government forces and civilians alternatively, depending on the strength and willingness of the Nigerian military and its allies to fight. In other words, the contrasting evolution of the daily event rates of the insurgents and government forces is explained by changes in the military strategies of the Nigerian army, by far the largest in the region.

From 2010 to 2015, Boko Haram was equally active in fighting the military and attacking civilians, a situation that reflected both the weakness of government forces and its inability to protect local populations effectively. The growing involvement of Nigerian forces since 2015 has forced Boko Haram to respond more aggressively to the military. As a result, 1.8 armed clashes were recorded for each attack against civilians. The peak in government attacks against the insurgents was reached in early 2015 following the MNJTF intervention. This major effort was followed by a series of military operations that progressively exhausted the fighting capability of Nigerian armed forces without eliminating Boko Haram and ISWAP permanently from the region. In 2016, for example, the Nigerian military launched two operations (Crackdown and Rescue Final) to destroy insurgents and rescue hostages in the Sambisa forest and two other operations (Karya Gwuia and Hard Knock) against Boko Haram camps in southern Borno State and the Lake Chad region. In 2017 and 2018, the Nigerian Army renewed its efforts against the stronghold of the Sambisa forest (Operation Deep Punch I) and the Lake Chad basin (Deep Punch II and Last Hold) [8].

The Nigerian military has gone on the defensive in recent years. This allowed Boko Haram and ISWAP to expand in several rural regions of northern Nigeria and continue their attacks against civilians and government forces. Soldiers stationed in the countryside lacked the firepower and military training necessary to fight insurgents successfully, and, as a result, their forward outposts were systematically overrun. Mobile patrols were also increasingly targeted by the insurgents, making outposts even more challenging to support in case of attack. As troops’ morale and willingness to fight insurgents quickly declined, the Nigerian military announced in 2019 that its soldiers would be pulled back from their forward posts and gathered in fortified camps. Nigerian troops and thousands of civilians were gathered in “super camps” inside garrison towns such as Bama or Monguno, leaving Boko Haram and ISWAP to fill the void left by government forces in the countryside [76]. Today, the daily rate of attacks by the government is well below that of Boko Haram and ISWAP and declining. The lowest daily rate of attacks against Boko Haram and ISWAP was reached in 2019 when the government decided to withdraw from the countryside.

From a tactical perspective, a noticeable change is the increasing use of suicide attacks by both Boko Haram and ISWAP between 2015 and mid-2018, with more than one event per week. Before Boko Haram conducted its first suicide attack in Abuja in June 2011, this tactic was virtually unknown in Nigeria. The use of suicide bombers in the country is explained by some observers by the influence of more globalised violent extremist organisations such as Al-Qaeda and Al-Shabaab [77]. Boko Haram has capitalised on other extremist organisations’ training and experience to develop this technique that now involves female suicide bombers [78, 79]. In the Lake Chad region, suicide bombings committed since 2011 have overwhelmingly targeted civilians (76%) rather than government buildings and military forces (6%) and have primarily been concentrated in Nigeria, where 80% of all fatalities are recorded. Yet, from early 2018, the number of suicide bombings has decreased to a minimal number by 2020.

### Constructing intervals and breaking points

**Fig 7:**
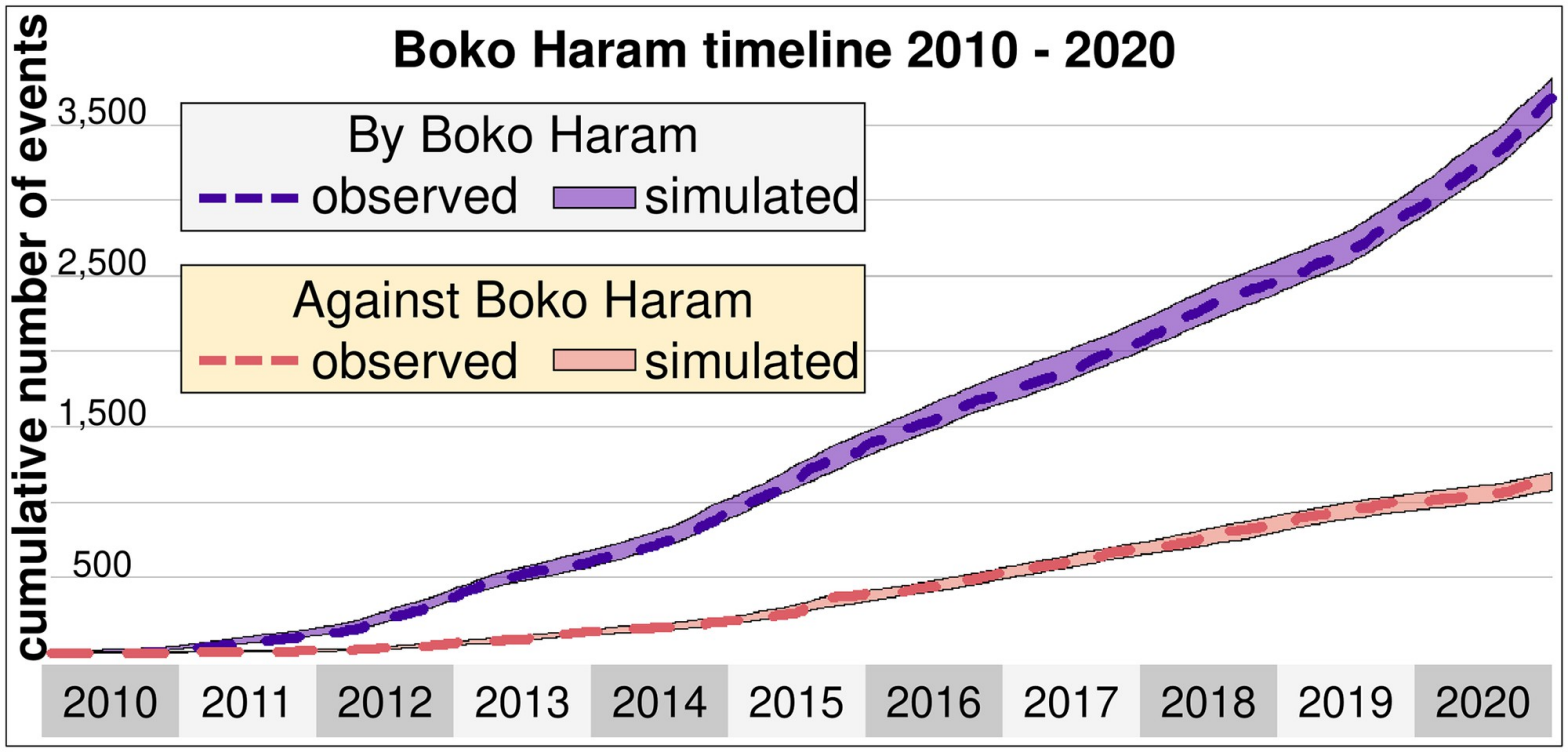
Uncertainty intervals of the model. Simulated intervals for the cumulative number of events committed by Boko Haram and ISWAP (blue) and against the terrorist group (pink) between 2010 and 2020. The observed number of events is the dashed line inside the corresponding intervals.

Taking into account the daily rate of events committed by and against Boko Haram and ISWAP, results show that the simulated intervals are a narrow band, with departures that are roughly 5% of the expected number of events (Fig 7). Furthermore, the observed number of cumulative events falls inside the obtained intervals. Thus, the obtained values of the daily rate λ(*k*) capture the daily fluctuations in how Boko Haram and ISWAP commit events.

Between two and eight breaking points, the most extended segment has at least 360 days, corresponding to an estimated rate of nearly a year. Between 9 and 13 breaking points, the smallest interval has at least 29 days, which means that our results estimate the daily rate for a month. Beyond 13 breakpoints, the segments are smaller. Seven shocks in the daily rate of events by Boko Haram are identified, the first in September 2010 and the latest in November 2019 (Table 4).

**Table 4:** Shocks of events committed by Boko Haram.

date	daily rate before	daily rate after	% change
10/09/2010	0.10	0.12	+21%
14/10/2010	0.16	0.20	+24%
7/10/2011	0.30	0.37	+22%
10/01/2012	0.40	0.49	+22%
9/02/2012	0.55	0.67	+22%
6/11/2018	1.09	1.32	+21%
29/11/2019	1.84	2.25	+23%

No shock was identified for the events committed by Boko Haram between February 2012 and November 2018, although the rate was not constant. Instead, it means that the transitions were relatively smooth and progressive. Also, eleven shocks are identified in the events committed against Boko Haram and ISWAP, three of which reduce the daily before-and-after rate of events (Table 5).

**Table 5:** Shocks of events committed against Boko Haram.

date	daily rate before	daily rate after	% change
19/05/2011	0.02	0.02	+24%
5/01/2012	0.07	0.08	+22%
30/01/2012	0.09	0.11	+29%
8/05/2013	0.20	0.24	+20%
20/01/2015	0.43	0.53	+23%
3/02/2015	0.57	0.69	+21%
1/04/2015	0.71	0.55	-22%
9/04/2015	0.55	0.43	-22%
6/03/2017	0.47	0.57	+22%
16/03/2017	0.64	0.51	-21%
7/11/2019	0.32	0.40	+26%

There are other methods for detecting shocks, although many of them are based on the assumption that the underlying distribution has some Gaussian noise [80, 81]. In the case of a discrete distribution, including daily counts such as the terrorism time series, the techniques are more limited. We validate our results with the E-Agglomerative algorithm for change point that gives a divisive hierarchical estimation algorithm for multiple change point analysis [82]. We detect an overlap in roughly 70% of the detected change points for the different time series we analysed, confirming that there are moments where the point process has changed its daily rate.

**Fig 8:**
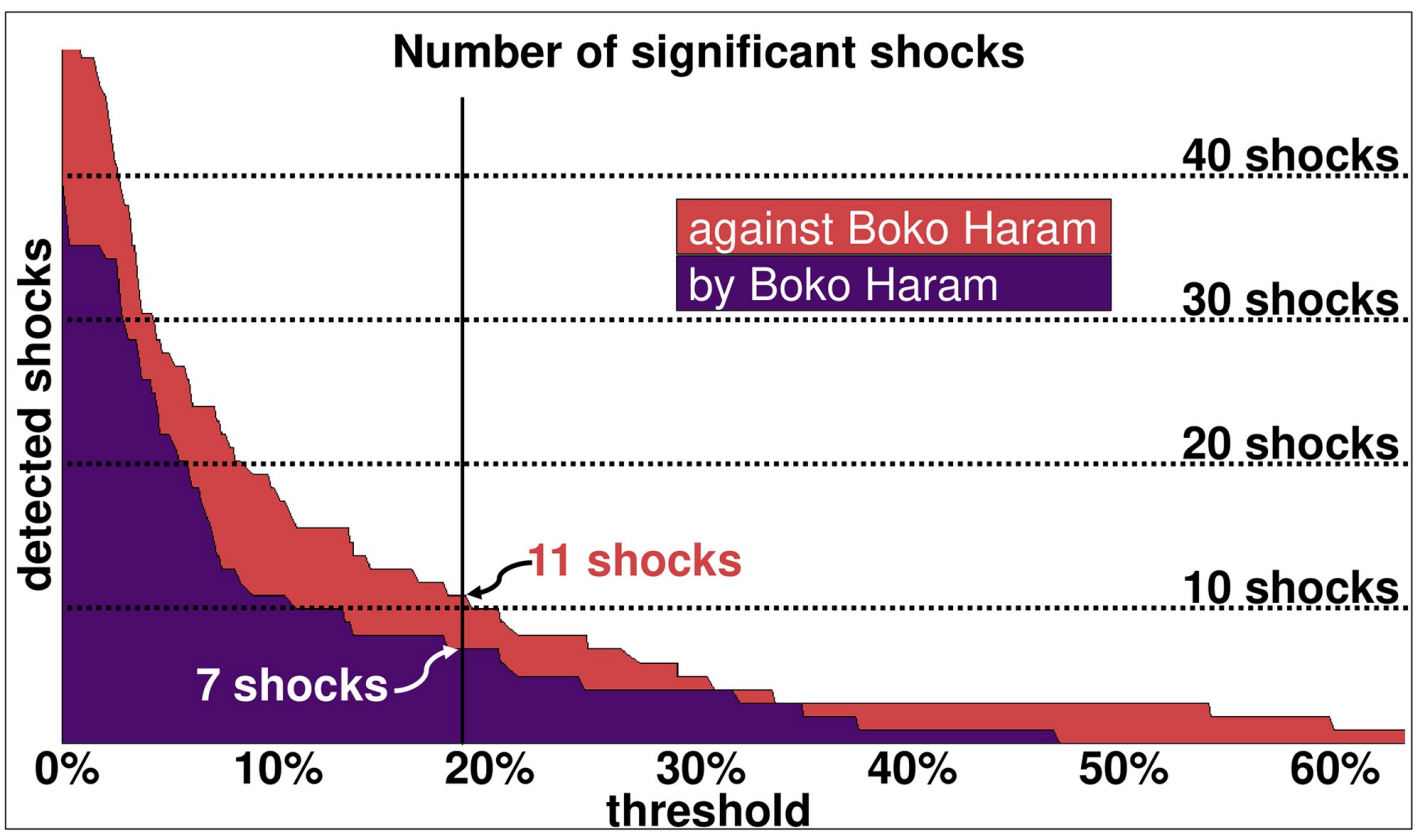
Shocks obtained. Number of shocks (vertical axis) as a function of the threshold between the before and after daily rates (horizontal axis).

The number of shocks depends on the threshold accepted as a jump in the seven-day moving average of the before-and-after rates. With a small threshold, relatively small shocks are detected, with up to 60 shocks for the events against Boko Haram and more than 40 for the events by Boko Haram (Fig 8). With a higher threshold, fewer -but more impactful- shocks are detected. With a threshold of 40% in the before and after rates, only one shock is detected in the daily rate of events by Boko Haram and three shocks in the daily rate of events against it.

### An application to Al-Shabaab events

Al-Shabaab (“The Youth” in Arabic) is a radical extremist organisation affiliated with Al-Qaeda whose political goal is to overthrow the Somali government and establish a new societal model based on a strict interpretation of Islamic law in the Horn of Africa [83]. Founded in late 2006 as a splinter group of the Islamic Courts Union (ICU), Al-Shabaab has become one of the most active militant groups in Africa [84]. The organisation has been involved in more than 14,000 attacks against the Somali government and the African Union Mission in Somalia (AMISOM), killing more than 35,000 people, according to ACLED. While Al-Shabaab has perpetrated spectacular attacks in neighbouring Kenya, most of the violent events and fatalities involving Al-Shabaab since the mid-2000s are located in Somalia (>95%).

**Fig 9:**
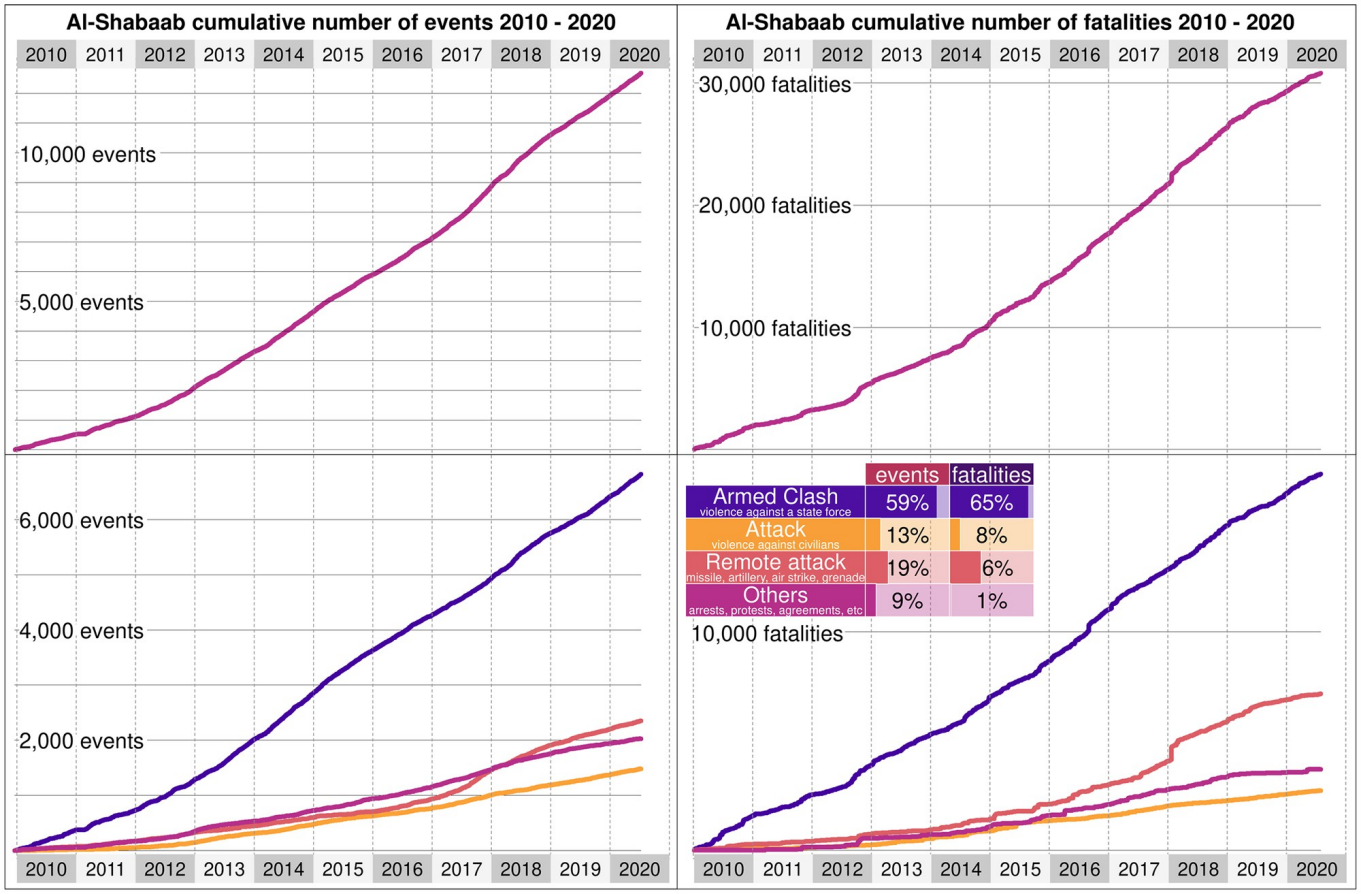
Events committed by and against Al-Shabaab. Left—Cumulative number of events (vertical axis) in which Al-Shabaab have participated since 2010 (horizontal axis), including both events by Al-Shabaab and against them. The top panel shows all events. The bottom panel shows events by type, including battles, violence against civilians, remote violence, and other events, such as protests or arrests. Right—Cumulative number of fatalities (vertical axis) in which Al-Shabaab have participated since 2010 (horizontal axis), including both events by Al-Shabaab and against them. The top panel shows all fatalities. The bottom panel shows events by type, including battles, violence against civilians, remote violence, and other events, such as protests or arrests.

We apply our technique to the Al-Shabaab events to test our method in a separate case study. The first Al-Shabaab event recorded in the ACLED database happened in August 2006, and more than 13,000 events have occurred since then (Fig 9). On average, there are 2.7 events each day. We test whether a homogeneous rate λ_0_ = 2.7 works for fitting the data. By June 2012, there were 2,129 events, but with a homogeneous rate, we expect between [*S*_*u*_(*t*), *S*_*l*_(*t*)] = [5537, 5833] events. Thus, we reject a uniform event rate. Like Boko Haram, the data shows that Al-Shabaab is becoming stronger, with nearly four events each day in the past year but only one event each week during their first year of operations. Having rejected a homogeneous rate, we follow our procedure and look at the cumulative curve (as in Fig 1).

**Fig 10:**
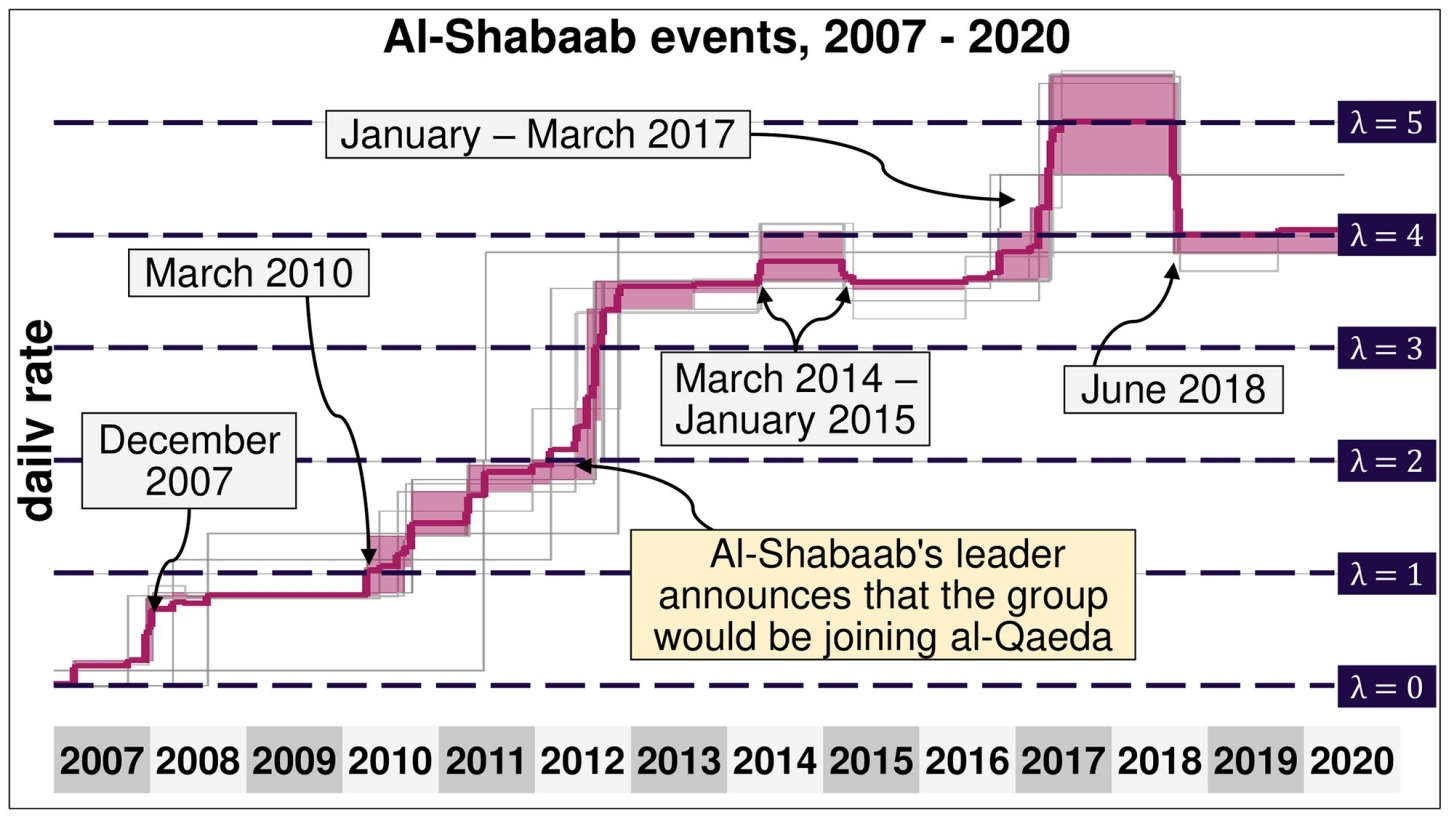
Rate of the different events related to Al-Shabaab. Daily rates in which Al-Shabaab takes part in different types of events, including attacks against civilians, against a state force, remote attacks and suicidal bombs, between 2007 and 2020. Some shocks were observed in their daily rate of events in December 2007; March 2010; February 2012; January 2017, and June 2018.

We obtain *κ* = 16 breaking points (Fig 10). After December 2007, Al-Shabaab participates in roughly 0.8 events daily, and this rate has been mostly stable for over two years. In February 2012, Al-Shabaab pledged allegiance to Al-Qaeda, which corresponds precisely to a shock that increased the daily number of Al-Shabaab events by nearly 75%. From 2012 to 2017, Al-Shabaab had roughly the same rate of 3.6 daily events with more than nine casualties daily. During this period, the terrorist group was heavily targeted by AMISOM, withdrew from the capital of Mogadishu, and largely avoided major battles. In early 2017 there was a shock when the daily number of Al-Shabaab events considerably increased from less than four events each day to more than five. For nearly a year, Al-Shabaab had its most intense and violent episode, with more than 37 events and 92 casualties each week. This period corresponds to a major resurgence of the group, which conducted numerous suicide attacks in Somalia and Kenya and considerably expanded its influence in rural areas. In total, Al-Shabaab has participated in more than 21 events each week since late 2012, with more than 50 weekly casualties.

## Conclusions

The objective of this article was to design a new approach to detect local trends and abrupt changes in the temporal distribution of terrorist events. Unlike other types of data for which thousands of daily observations are available, terrorist events have low frequencies and are usually sparse in time, which makes most time-series techniques unsuitable. Our approach is based on fitting a piecewise linear function to the cumulative events and analysing the corresponding gradients and breaking points from such regressions. This iterative method considers multiple breaking points, from which rate outliers can be dropped, and produces several intervals that detect potential departures from the rates. For temporal data, it detects long-term trends and shocks rather than local shifts.

The outcome of our approach is a daily rate λ(*t*). The procedure can be applied to all events or specific types, such as battles between government forces and terrorist organisations, attacks against civilians, or more remote attacks, such as suicide bombings. Although the methodology might produce many minor shifts in the daily rate of events, shocks are detected by considering only significant jumps in the daily rates. Our analysis detects several breaking points in the distribution of violent events that correspond to internal and external change factors for terrorist organisations. The case of Boko Haram and ISWAP reveals that significant offensives, such as the one launched by the Nigerian government and its regional allies and the global allegiance to terrorist groups, correspond to essential shocks. We identify similar trends in the recent evolution of Al-Shabaab, suggesting that our approach can be generalised to other significant insurgencies.

To our knowledge, our analysis is the first to consider the directionality of attacks to understand who is ultimately responsible for increasing violence in a particular region. By identifying the perpetrator and the target of an attack, our work opens new avenues of research for conflict analysts interested in the expansion of terrorist organisations in Africa. In the Lake Chad region, for example, our work suggests that Boko Haram and its splinter group are responsible for nearly 70% of the violent events recorded since 2010. Even more worrying, Boko Haram and ISWAP seem to have recovered rapidly from the 2015 offensive led by the Nigerian government and the MNJTF. In recent years, both organisations have been able to increase the number and frequency of their attacks. Boko Haram and its splinter group concentrated their attacks against military forces until 2018, a strategy that eventually led the Nigerian military to dismantle its forward outposts, withdraw from rural regions and concentrate its troops and some civilians in garrison towns. This strategy has led to a substantial decrease in the number of casualties in the rank of the Nigerian military and the number of attacks conducted against Boko Haram and ISWAP. Since 2019, the two main factions of the Jihadist organisation have rapidly filled the void left by the government and resumed their offensive against civilians outside fortified camps. As a result, the daily rate of attacks against Boko Haram has remained low and represents only 15% of the daily rate of Boko Haram and ISWAP events.

These findings have policy implications. In the Lake Chad region, they suggest that the current strategy to concentrate military forces in fortified “super camps” has largely failed to restore political stability and protect civilian populations. Withdrawing military troops from rural areas may help restore the morale and fighting ability of the government army, but it also leaves millions of civilians unprotected in rural areas. Civilian populations in fortified camps are also increasingly cut from their livelihoods and may become permanent refugees. This shift mirrors the Strategic Hamlet Program developed by the governments of South Vietnam in the 1960s. It contradicts one of the most fundamental principles of counterinsurgency warfare [85], which is to place government forces as close as possible to the civilian population to separate them from the insurgents and provide them with all the protection necessary for their collaboration with the government.

Our study has some limitations. First, some events might be missing, the number of casualties or the date might not be precise, and our method to detect directionality is imperfect. More detailed and precise data would help improve our understanding of terrorist groups and why we observe steady trends and sudden shocks. In the Supplementary information, we added a comparison of our results using the Global Terrorism Database (GTD), which shows that the most significant difference between the two datasets is that ACLED considers all events (including those against a group) whilst GTD considers only events committed by the group [7, 86]. Second, further attempts to study the temporal evolution of terrorist organisations should also consider that it is impossible to estimate the real impact of each event on the daily lives of civilian populations and the military capabilities of state and non-state actors. Events without casualties may significantly impact state forces or insurgents, for example, if a terrorist cell is dismantled or some infrastructure is damaged. To assess the direct impact of the conflict, a more comprehensive approach should also consider injuries and infrastructure damage, shifts in public opinion, fear related to future events or even the size of the displaced population. Finally, our approach depends on past events; therefore, predictions should be considered cautiously. While there is some stability in the daily number of events committed by or against the group, predicting the future number of events is possible with the most current rates, but only briefly. The organisations studied in the paper suggest that the daily rate of events is susceptible to experiencing sudden increases and decreases in the intensity of events.

## Supporting information

S1 File (PDF)

S2 File (PDF)
