# Supplementary material for: Detecting trends and shocks in terrorist activities
Source: PLoS One. 2023 Sep 15;18(9):e0291514. doi: 10.1371/journal.pone.0291514 (PMC10503774; doi:10.1371/journal.pone.0291514)
Supplement: S1 File — (PDF) [file pone.0291514.s001.pdf]

Detecting trends and shocks in terrorist activities Supporting information

Rafael Prieto-Curiel[1*], Olivier Walther[2], Ewan Davies[3],

1 Complexity Science Hub, Josefstädter Str. 39, 1080 Vienna, Austria
2 Department of Geography, University of Florida, Gainesville, 32611, Florida, USA
3 Mathematical Institute, University of Oxford, Radcliffe Observatory Quarter, OX2 6GG, Oxford, UK

* prieto-curiel@csh.ac.at

Considering other data sources

Detecting significant shocks depends on four key elements:

- The data considered to analyse a terrorist group.
- Detect whether a homogeneous rate and the natural variability of a discrete variable are enough to explain the observed fluctuations in the number of events.
- If the homogeneous rate is rejected, the results depend on the number of modelled shocks.
- After combining the rates into a single intensity, the results depend on a parameter to define when jumps are considered a shock.

Here, we analyse the impact of considering other data sources and the relevance of considering a varying number of shocks.

Events related to Boko Haram can be analysed using several sources. We have used ACLED as the primary data source [1]. However, the Global Terrorism Database (GTD) is also frequently used to analyse terrorism worldwide [2]. GTD data has relevant additional variables to analyse events, such as weapon types, attacks, and targets.

To verify that our results were not due to the subjective choice of the source, we investigated the cumulative number of fatalities related to Boko Haram in the ACLED data and GTD (S1 Fig.). The GTD dataset registers six categories of terrorist incidents: Armed Assault, Assassination, Bombing/Explosion, Facility/Infrastructure Attack, Hijacking, and Hostage Taking. Therefore, the GTD does not consider all events related to some terrorist group, but only events committed by them. ACLED adopts a more comprehensive definition of political violence, including battles, arrests, protests or riots, which is particularly relevant to our study. This difference is relevant since ACLED has a broader range of events related to a terrorist group. ACLED records more than 5,000 violent events associated with Boko Haram and more than 40,000 casualties until March 2020. During the same period, GTD had 2,500 events and only 20,000 deaths. Therefore, GTD has captured roughly half of the ACLED events, and all of them would be considered committed by the group and not against it. GTD only captures events committed by a terrorist group and not against them, considerably limiting the study of directionality.

Boko Haram events registered in the GTD and ACLED dataset.

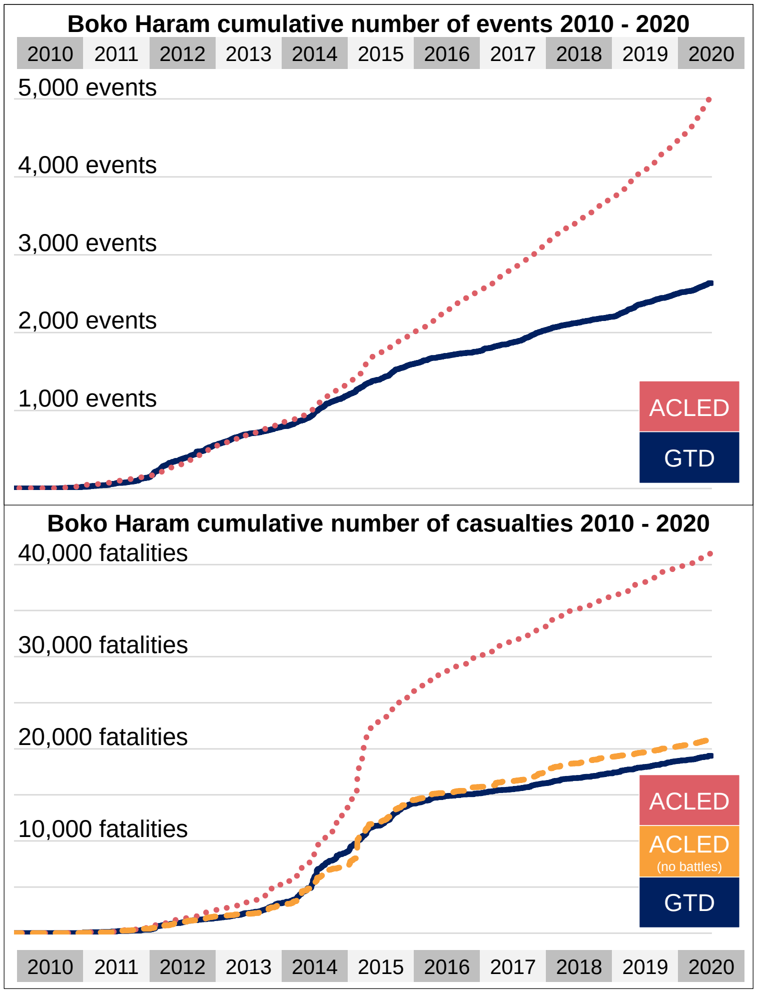

S1 Fig. Cumulative number of events (top) and casualties (bottom) related to Boko Haram registered in the GTD dataset and the ACLED dataset. In the case of ACLED, we also consider the case without battles.

For ACLED, we also compared the cumulative fatalities when battles were not considered (S1 Fig.). By not including the battles in the ACLED dataset, more than half of the casualties related to Boko Haram are not included. Indeed, most battles in the ACLED dataset are missing events in GTD since they would not be considered "terrorism". The three cumulative curves (GTD, ACLED and ACLED with no battles) have a similar shape but have a different scale, with a rapid increase around 2015. Particularly when battles are removed, ACLED and GTD have a similar cumulative shape fundamentally, meaning that both systems are capturing a similar increase in the intensity of Boko Haram events. The daily rate for the GTD and ACLED without battles is nearly the same, except for a few minor jumps in 2014 and 2017. In the case of Boko Haram, we obtain similar results regarding the daily intensity when we consider the number of GTD events and the number of ACLED events ignoring battles.

References

1. Raleigh C, Linke A, Hegre H, Karlsen J. Introducing ACLED: an Armed Conflict Location and Event Dataset: special data feature. Journal of Peace Research. 2010;47(5):651–660.

2. Global Terrorism Database 1970 2020 G. National Consortium for the Study of Terrorism and Responses to Terrorism; 2022. Available from: https://www.start.umd.edu/gtd.
