# Supplementary material for: Detecting trends and shocks in terrorist activities
Source: PLoS One. 2023 Sep 15;18(9):e0291514. doi: 10.1371/journal.pone.0291514 (PMC10503774; doi:10.1371/journal.pone.0291514)
Supplement: S2 File — (PDF) [file pone.0291514.s002.pdf]

Detecting trends and shocks in terrorist activities
Supporting information

Rafael Prieto-Curiel[1*], Olivier Walther[2], Ewan Davies[3],

1 Complexity Science Hub, Josefstädter Str. 39, 1080 Vienna, Austria
2 Department of Geography, University of Florida, Gainesville, 32611, Florida, USA
3 Mathematical Institute, University of Oxford, Radcliffe Observatory Quarter, OX2 6GG, Oxford, UK

* prieto-curiel@csh.ac.at

Varying the number of shocks

We observed that a homogeneous rate does not explain the fluctuations observed in the number of Boko Haram events, so a constant λ is rejected. Then, we took a threshold of 20% change in the rate as the definition of a shock. Changing the threshold alters the number of shocks detected (Fig. 8 in the manuscript). Here, we analyse the impact of the second element, that is, the number of modelled shocks.

Having rejected a constant rate means that at least one shock is needed. When a single change is considered, the procedure finds the date, minimising the error. When two changes are considered, the procedure does not recursively find a second date after the one already encountered. Instead, it finds two separate dates to model the changes. This implies that the date encountered with one change might not match the dates with two shocks. For instance, in the number of Boko Haram events, 2013/11/14 is considered as the date for the change based on a single shock. When two changes are considered, 2013/02/23 and 2018/12/17 are the dates detected. Dates for the detected modifications are not a perfect match but suggest when abrupt changes could have occurred.

Similarly, when we increase the number of changes, we observe that the detected dates are not a perfect match of others (S1 Table). There are, however, some common patterns in the dates on which changes are detected. For example, in most cases, a change point during November 2019 is identified. Although the exact date varies slightly, it highlights that likely a shock occurred around those days, regardless of the number of changes considered.

Also, notice that the dates vary substantially for a few changes. This is because analysing the daily rate of events for over ten years has some evident fluctuations that cannot be accurately captured by only two or three different rates, as in the case of one or two changes.

Boko Haram breaking points.

shocks	date
1	2013/11/14
2	2013/02/23 2018/12/17
3	2011/10/14 2014/02/02 2019/01/15
4	2011/10/03 2014/05/17 2015/04/05 2018/12/13
5	2010/10/16 2012/02/08 2013/02/09 2013/11/24 2019/01/16
6	2011/10/03 2014/05/17 2015/04/15 2016/04/22 2016/11/06 2019/01/23
7	2010/10/16 2012/02/08 2013/01/23 2014/03/30 2015/04/08 2018/11/05 2019/11/27
8	2010/10/16 2012/02/08 2013/01/25 2014/03/20 2015/08/13 2017/05/29 2017/11/14 2018/12/14
9	2010/10/16 2012/02/08 2013/01/23 2014/03/31 2015/04/16 2016/04/21 2016/10/31 2018/12/08 2019/11/28
10	2010/10/16 2012/02/07 2013/02/23 2013/08/26 2014/04/18 2015/04/14 2016/04/22 2016/10/31 2018/12/08 2019/11/28
11	2010/10/09 2012/01/06 2012/08/02 2012/12/24 2014/03/30 2015/04/17 2016/03/05 2017/05/03 2017/11/28 2018/11/03 2019/11/27
12	2010/10/09 2012/01/06 2012/07/31 2013/01/12 2013/09/05 2014/04/17 2015/04/15 2016/03/05 2017/05/03 2017/11/28 2018/11/03 2019/11/27
13	2010/09/09 2011/05/13 2012/02/18 2013/02/20 2013/08/18 2014/02/07 2014/04/30 2015/04/14 2016/03/06 2017/05/03 2017/11/28 2018/11/03 2019/11/27
14	2010/09/09 2011/05/12 2012/02/18 2013/02/21 2013/08/25 2014/04/06 2015/02/12 2015/02/25 2016/04/26 2016/10/23 2017/06/12 2017/11/24 2018/11/03 2019/11/27
15	2010/10/01 2011/10/04 2012/01/25 2012/08/04 2013/01/11 2013/09/05 2014/04/17 2015/04/14 2016/04/29 2016/05/02 2016/10/24 2017/06/12 2017/11/24 2018/11/03 2019/11/27

S1 Table. Observed breaking points when a different number of shocks is considered in the daily rate of Boko Haram events.
